# Severe Thrombocytopenia in a 30-Year-Old African American Male With Newly Diagnosed Sarcoidosis: A Case Report

**DOI:** 10.7759/cureus.34135

**Published:** 2023-01-24

**Authors:** Melisa Pasli, Katie K Lovell, Sai Swarupa R Vulasala, Marsha L Hairr, Revanth Reddy Bandaru, Mohammad Z Khalilullah, Leonard Johnson

**Affiliations:** 1 Medical School, Brody School of Medicine at East Carolina University, Greenville, USA; 2 Internal Medicine, East Carolina University Health, Greenville, USA

**Keywords:** itp management, clinical hematology, thrombocytopenia, immune thrombocytopenia (itp), sarcoidosis

## Abstract

Sarcoidosis is a multisystem inflammatory disorder characterized by the formation of non-caseating granulomas. Hematological manifestations such as thrombocytopenia are unusual presentations of the disease. Various theories have been proposed for the development of thrombocytopenia in patients with sarcoidosis such as decreased production in bone marrow caused by granuloma formation, hypersplenism, and immune thrombocytopenia (ITP). We present a case of a 30-year-old African American male with ITP secondary to sarcoidosis who presented with a sudden onset of buccal mucosa and mucocutaneous bleeding and was found to have severe thrombocytopenia with values reaching as low as 1000/uL without prior history of easy bruising or bleeding. Overall, our patient had dyspnea, mucocutaneous bleeding, and was found to have mediastinal and hilar adenopathy, isolated thrombocytopenia, no splenomegaly, and non-necrotizing granulomas in the lymph nodes. The patient received platelet transfusions without initial response and received intravenous immunoglobulin (IVIG), romiplostim, and steroids with subsequent improvement in the platelet count after sufficient administration of a treatment regimen of approximately one week. Confounding factors that resulted in diagnostic uncertainty of our patient presentation included travel history with prophylactic antimalarial medications, doxycycline usage, only slightly elevated Angiotensin-Converting Enzyme (ACE) levels, and imaging features concerning metastatic disease vs. lymphoma. The clinical diversity of sarcoidosis often leads to diagnostic uncertainty and treatment delays due to its resemblance to other more common disorders. This is a novel case report of the earliest temporal presentation of severe thrombocytopenia and sarcoidosis in an African American male reported in the literature.

## Introduction

Sarcoidosis is a multisystem disorder characterized by the formation of non-caseating granulomas [1]. It affects multiple organ systems including, but not limited to, the lungs, joints, heart, eyes, and skin [2]. Pulmonary manifestations are common, with patients typically presenting with symptoms such as dyspnea or cough [2]. More than 90% of patients with sarcoidosis demonstrate pulmonary involvement [3]. Sarcoidosis is a diagnosis of exclusion made using clinical and radiological findings and further supported by histological identification of non-caseating granulomas [4].

Isolated thrombocytopenia is an uncommon presentation of sarcoidosis compared to other hematological abnormalities, such as anemia and leukopenia [1]. Three main pathophysiological hypotheses of thrombocytopenia in sarcoidosis are proposed: hypersplenism, bone marrow infiltration of granulomas, and immune thrombocytopenia (ITP) [5-7]. The first occurrence of thrombocytopenia in sarcoidosis was described by Jersild et al. in 1938, and the association between sarcoidosis and thrombocytopenia was primarily reported in retrospective studies with an incidence rate of 1-2% [5,8,9]. Though the inciting event in sarcoidosis is undiscovered, granulomas typically develop to restrict pathogens, confine inflammation, and preserve surrounding tissue. CD4+ T cells interact with antigen-presenting cells, thereby initiating the development and maintenance of granulomas, a cardinal feature of sarcoidosis. This chronic inflammation is hypothesized to stem from an exaggerated T-cellular- and humoral-mediated response to a presenting antigen that is not appropriately eradicated [10]. Alternatively, granuloma formation may predispose the patients to an autoimmune state [11]. Disturbances may be present in both T-cell and B-cell immunological responses, suggesting both a humoral and cellular component contributing to the pathogenesis of the disease [12]. Corticosteroids remain the first-line treatment in these patients, further validating the relationship between sarcoidosis and an underlying autoimmune etiology [7,12]. We present a case of severe thrombocytopenia, with values reaching as low as 1000/uL as a presenting feature of newly diagnosed sarcoidosis in a young African American male.

## Case presentation

A 30-year-old African American male with no significant past medical history presented with a sudden onset of buccal and mucocutaneous bleeding and severe thrombocytopenia with a platelet count of 4000 uL. He denied a history of fevers, weight changes, fatigue, malaise, chronic cough, palpable lymphadenopathy, prior skin rash, or bleeding dyscrasias. A review of systems was significant for shortness of breath that had been ongoing for two months and bleeding gums and rash that had started one day prior. Social history was notable for previous tobacco use (5 pack years) and current daily use of nicotine vape. Other pertinent positive history included recent travel to Ghana within the month before admission for which he received malaria prophylaxis with atovaquone-proguanil. After his return from Ghana, he developed dyspnea and was evaluated with a chest X-ray that showed bilateral hilar adenopathy. He was scheduled to see a pulmonologist for bronchoscopy and, at that time, he was prescribed doxycycline 100 mg twice a day and prednisone 10 mg daily. However, his condition deteriorated due to acute onset bleeding, which prompted him to seek medical attention.

**Figure 1:**
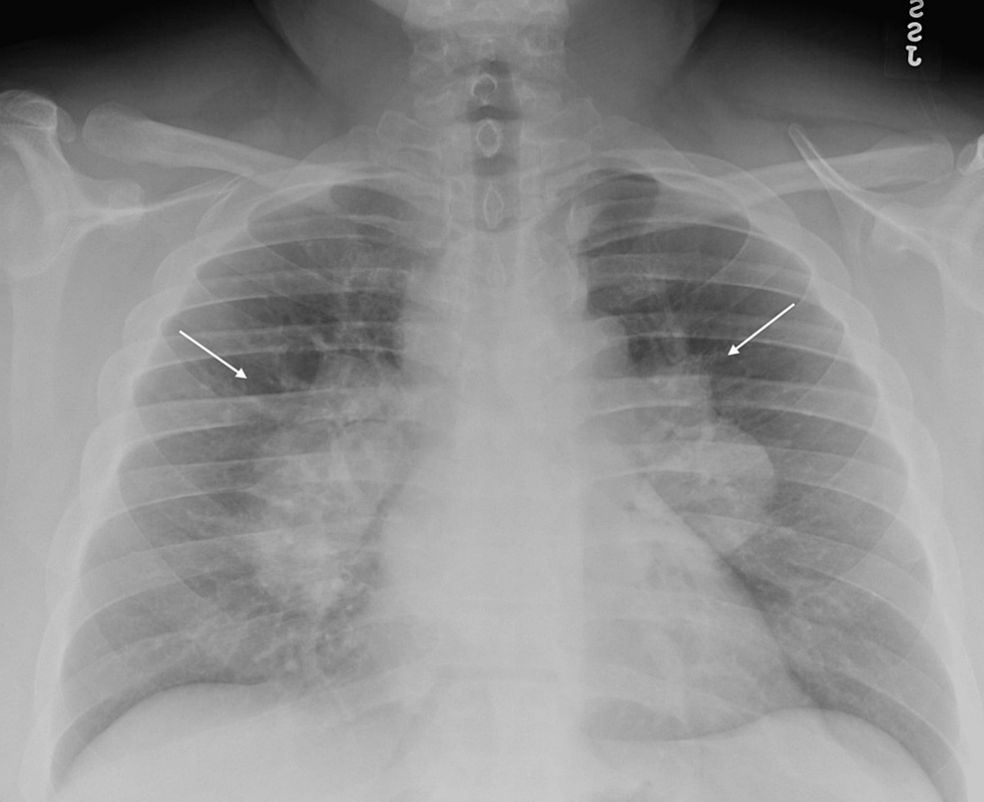
Chest X-ray The image is demonstrating severe mediastinal and bilateral lymphadenopathy.

At the time of admission, he was afebrile (37.1°C) with a pulse rate of 90 beats per minute (bpm) and elevated blood pressure of 140-150/90-100 mm Hg. He was alert and oriented, able to answer questions and no meningeal signs were present. Erythematous oral mucous membranes and diffuse petechiae were noted in the oral cavity. His chest was clear to auscultation, and he had a regular heart rhythm and rate. There was no hepatosplenomegaly. No petechial spots were noted on extremities. On admission, fibrinogen levels were 382 mg/dL (ref 200-400 mg/dL), HIV and Hepatitis panels were negative, and folate and vitamin B12 levels were within normal limits (9 ng/mL and 259 pg/mL, respectively). A review of the peripheral smear showed thrombocytopenia. A rare plasma cell and nucleated red blood cell (RBC) precursors were noted. There was no overt increase in blasts, and no large atypical lymphocytes, schistocytes, or significant granulocytic dysplasia was observed. Additional admission laboratory values found are given in Table 1. Further diagnostics included a chest X-ray (Figure 1) which demonstrated significant bilateral hilar and mediastinal lymphadenopathy.

**Table 1:** Admission and discharge laboratory values of laboratory tests Platelet count (Plt), hemoglobin (Hgb), white blood count (WBC), hematocrit (Hct), prothrombin time (PT), partial thromboplastin time (aPTT), lactate dehydrogenase (LDH), angiotensin-converting enzyme (ACE) level, C-reactive protein (CRP) and erythrocyte sedimentation rate (ESR)

Test	Admission Values	Discharge Values	Reference Range
Platelet Count (Plt)	4	136	150-440 k/uL
Hemoglobin (Hgb)	13.2	11.9	13.0-18.0 g/dL
White blood count (WBC)	6.01	2.93	4.50-11.00 k/uL
Hematocrit (Hct)	37.8	35.6	35-47%
Prothrombin Time (PT)	13.8	13.7	10.2-12.9 seconds
Activated Partial Thromboplastin Time (aPTT)	35.7	26.5	25.1-36.5 seconds
Lactate Dehydrogenase (LDH)	250		125-220 U/L
Angiotensin-Converting Enzyme (ACE) Level	83		16-85 U/L
C-Reactive Protein (CRP)	15.3		<5 mg/L
Erythrocyte Sedimentation Rate (ESR)	34		<15 mm/hr

**Figure 2:**
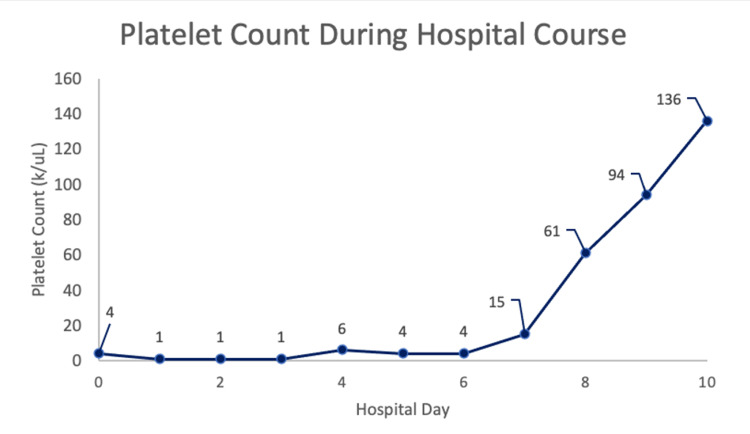
Graph demonstrating platelet count throughout the hospital course

Based on the decreased platelet count with an otherwise unremarkable complete blood count (CBC), a preliminary diagnosis of ITP was made with differentials including drug-induced causes (doxycycline, atovaquone-proguanil), autoimmune causes, malignancy, lymphoma, and sarcoidosis. Due to severe thrombocytopenia, the patient received two units of platelets in the emergency department and was admitted for further evaluation. Despite transfusion, the patient’s platelet count subsequently declined to 1000 uL. Steroids are the first-line therapy for ITP; however, this therapy was withheld in our patient due to suspicion of mediastinal lymphoma and because steroid treatment before biopsy may cause a delay in definitive diagnosis and adversely affect pathological accuracy. By hospital day 2, the patient had received five units of platelets, and his platelet count remained at 1000uL. After consultation with hematology, the patient was given 1 g/kg of intravenous immunoglobulin (IVIG) (100g/day), and a bronchoscopy with a lymph node biopsy was planned once the patient’s platelet count reached 10,000 uL. The patient’s platelet count plateaued at 1000 uL on hospital days two and three despite receiving seven total platelet transfusions. He was then given a second-line therapy with romiplostim (Nplate®) at 5mg/kg to stimulate platelet production. Platelet count improved to 6000 uL on hospital day four. A graph of platelet levels is presented in Figure 2. 

**Figure 3:**
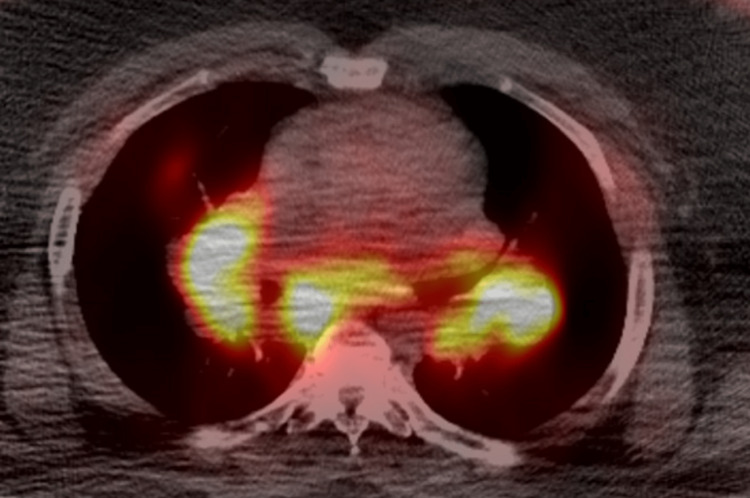
Chest positron emission tomography (PET) scan The image is demonstrating multiple hypermetabolic adenopathies of the hilar and mediastinal lymph nodes.

**Figure 4:**
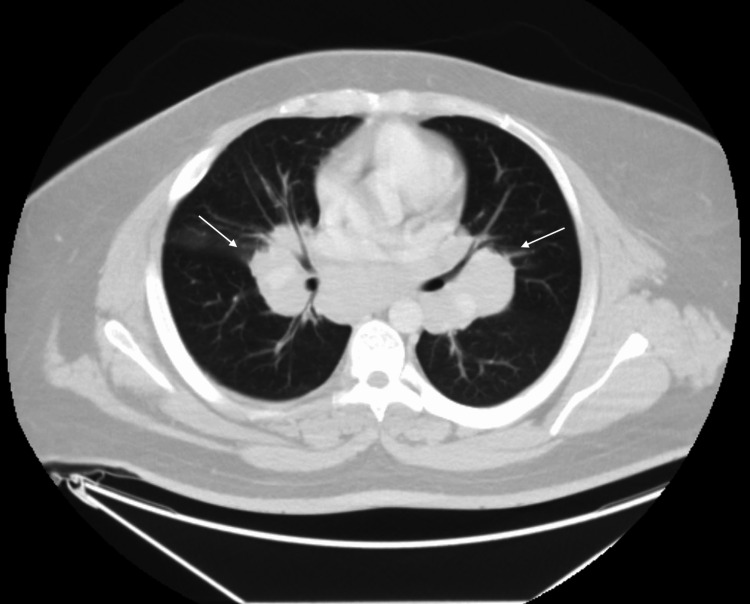
Chest computed tomography (CT) scan The image is demonstrating bulky diffuse mediastinal and hilar lymphadenopathy measuring up to 3.8 cm within the subcarinal space.

He was further evaluated with a positron emission tomography (PET) scan and computerized tomography (CT) scan of the chest (Figures 3,4 respectively) and underwent CT-guided bone marrow biopsy and bronchoscopy/lymph node biopsy.

**Figure 5:**
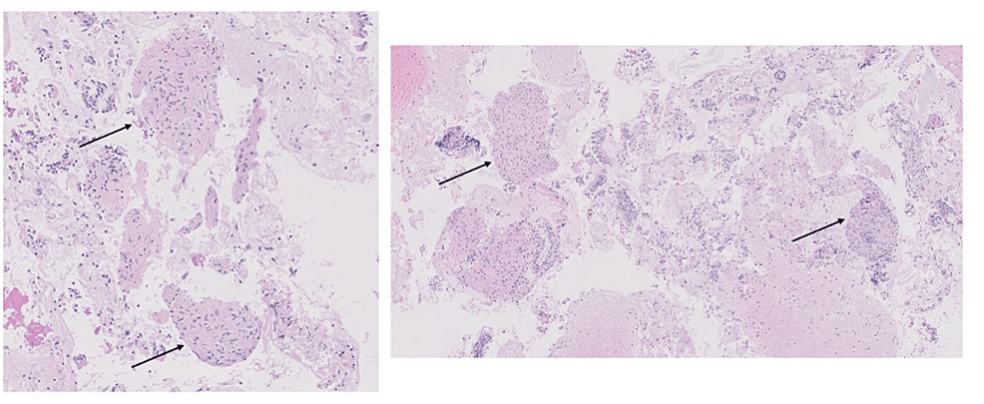
Pathology images (5x) Pathology images show non-necrotizing granulomatous inflammation. The lymphoid tissue population was identified, consistent with a lymph node sampling. No cells diagnostic of malignancy were identified.

**Figure 6:**
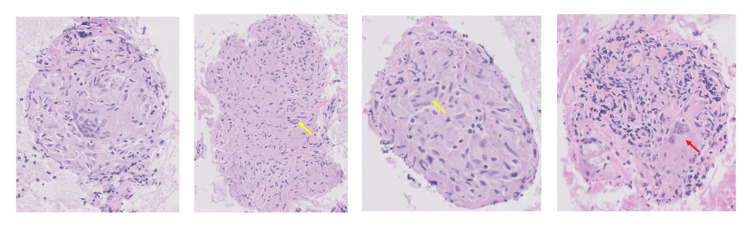
Pathology images (20x) Pathology images show multiple non-necrotizing granulomas composed of aggregates of epithelioid histiocytes with multinucleate giant cells of the Langhans type (yellow arrows) and foreign-body type (red arrow), and lymphocytes. Grocott methenamine silver (GMS) and acid-fast bacilli (AFB) stains are negative.

Bone marrow aspirate demonstrated normocellular marrow with megakaryocyte proliferation, which is consistent with an immune destructive process such as secondary ITP, and there was no definitive evidence of marrow involvement by non-Hodgkin lymphoma (NHL) or metastatic carcinoma. The findings did not meet the criteria for myelodysplastic syndrome based on morphological assessment. Cytogenetic analysis showed a normal male karyotype. Histopathological examination of lymph node biopsy demonstrated non-caseating granulomatous inflammation, and cells diagnostic of malignancy were not identified (Figures 5, 6).

He was diagnosed to have developed ITP, most likely secondary to sarcoidosis, started therapy with prednisone 60 mg daily, and subsequently improved in platelet count. His cell counts were white blood cell (WBC) 2.93 k/uL (lo), hematocrit 35.6% (lo), and platelets 136k/uL at the time of discharge.

## Discussion

Immune thrombocytopenia is defined as isolated thrombocytopenia with platelet count < 100 x10^9^ k/uL on at least two occasions in the absence of drug-induced thrombocytopenia, hypogammaglobulinemia, splenomegaly, portal hypertension, or pancytopenia [13]. While sarcoidosis onset often predates ITP onset by an average of 48 months [13], our patient had a recent workup for sarcoidosis a month before ITP presentation. In a study by Mahévas et al., the average onset of sarcoidosis before ITP onset was 6-216 months, but five of the 20 patients in the study had a simultaneous presentation of ITP and sarcoidosis [13]. Our patient presentation was further complicated by recent travel and subsequent prophylactic therapy, which could have triggered drug-induced thrombocytopenia. Thus, despite prior workup for sarcoidosis, the unique presentation necessitated testing to rule out all other causes of his thrombocytopenia including malignancy and drug-induced causes.

As with sarcoidosis, first-line therapy with systemic steroids and, occasionally, second-line therapies like IVIG, vincristine, disulone, danatrol, hydroxychloroquine (HCQ), or rituximab are the standard of care for patients presenting with ITP secondary to sarcoidosis. In a retrospective study of twenty patients with concurrent sarcoidosis and ITP, twelve patients had a complete resolution of ITP with first-line glucocorticoid therapy, and seven required second-line therapies resulting in five of those patients reaching a complete resolution, one patient reaching a partial resolution with first-line and second-line therapy, and one patient without resolution despite first-line and second-line therapies. One other patient required no treatment for complete resolution [13]. In patients with chronic ITP after the failure of first-line therapies, romiplostim (a thrombopoietin {TPO} receptor agonist) is often used. Due to its adverse- effect profile such as bone marrow fibrosis, headache, and fatigue, it remains second-line therapy for patients with chronic ITP refractory to first-line agents [14]. During admission, our patient received one dose of romiplostim, IVIG (1g/kg), and, as needed, platelet transfusions with a moderate response before initiation of corticosteroids.

Eighty percent of patients with sarcoidosis present with extrathoracic involvement including ocular, liver, peripheral lymph nodes, and sinonasal [13]. While the association of thrombocytopenia with sarcoidosis is well documented, the presentation in practice is rare. Due to the rarity, there are no specific guidelines for the management of thrombocytopenia in patients with sarcoidosis [1]. Immune suppression with steroids is deemed the first-line treatment of thrombocytopenia with concurrent sarcoidosis [1] as steroids inhibit established autoimmunity and decrease granuloma formation in the bone marrow [12], which may enhance platelet count. Furthermore, immune suppression is often utilized in the treatment of both thrombocytopenia and sarcoidosis as both are associated with an autoimmune or inflammatory state, thus providing the dual-utility of the treatment regimen in a patient with ITP secondary to sarcoidosis. However, lymphoma had to be ruled out before our patient received regular steroids. The delayed platelet count response may have been expedited with an earlier introduction of regular steroid therapy, which likely would have reduced the patient’s cost burden.

We present our case as a novel addition to the current literature as only one other case in a young male at age 32 has been reported in the literature. Although the case report by Korogodina et al. [15] presented a similarly aged man with severe thrombocytopenia with similar values (3000/uL at presentation with a nadir of 1000/uL), the patient had super-imposed focal segmental glomerulosclerosis (FSGS) and a five-year history of sarcoidosis diagnosis. Conversely, our patient was younger and was newly diagnosed with sarcoidosis (symptom onset within the last month) without evidence of end-organ damage.

Most patients with pulmonary sarcoidosis may not need any therapeutic interventions, as one study demonstrated only 34.5% of patients needed glucocorticoid treatment [16]. Systemic therapy may be withheld due to the nature of the pathology, and sarcoidosis may undergo spontaneous regression in up to 80% of patients with stage one disease without inducing permanent end-organ damage [17]. Conversely, systemic therapy is deemed necessary for those with progressive disease exhibiting symptoms or lung function deterioration [17]. Systemic glucocorticoids are deemed first-line treatment but may have negative side effects such as hyperglycemia, cushingoid side effects, osteoporosis, cataracts, and psychosis [18]. Meta-analyses of 13 randomized placebo-controlled clinical trials (RCTs) have shown significant improvements in both pulmonary function and symptoms in many trials in those patients who received glucocorticoids [19]. As such, our patient was prescribed steroids following hospital discharge after his platelet levels had improved and stabilized with second-line therapies.

Our treatment plan is well supported by the currently established treatment algorithms. Publication of this case report will serve as a guideline for second-line therapies to stabilize a patient presenting with severe ITP that is likely secondary to sarcoidosis in the context of confounding factors which require further investigation and treatment of underlying causes before glucocorticoid administration.

## Conclusions

This case reports illustrates the earliest temporal presentation of severe thrombocytopenia and sarcoidosis in an African American man. The extensive workup as demonstrated in this case report illustrates the challenge associated with diagnosing ITP secondary to sarcoidosis. Conducting a complete blood count (CBC) earlier during the initial evaluation while awaiting bronchoscopy or during routine follow-up examinations may have prevented this hospital admission. Our patient had an extensive workup including a fluorodeoxyglucose (FDG)-PET scan to rule out malignancy; there was also a high suspicion of sarcoidosis despite its rare clinical association with ITP presentation. Further studies of thrombocytopenia and sarcoidosis are warranted to decrease hospital and resource utilization and to decrease cost in the workup of patients with thrombocytopenia secondary to sarcoidosis.
